# Research on the Efficiency of Green Agricultural Science and Technology Innovation Resource Allocation Based on a Three-Stage DEA Model—A Case Study of Anhui Province, China

**DOI:** 10.3390/ijerph192013683

**Published:** 2022-10-21

**Authors:** Sheng Yao, Guosong Wu

**Affiliations:** 1 Institute of Agricultural Economics and Information, Anhui Academy of Agricultural Sciences, Hefei 230031, China; 2 School of Economics and Management, Huzhou University, Huzhou 313000, China; 3 Institute of “Two Mountains” Theory, Huzhou University, Huzhou 313000, China

**Keywords:** science and technology innovation, resource allocation, Anhui Province, China

## Abstract

In order to achieve sustainable development of agriculture, people have gradually begun to attach importance to the development of low-carbon agriculture and to regard green agricultural technology innovation and promotion as increasingly more important. Taking the Anhui Province of China as an example, this study analyzed the impact of green agricultural science and technology innovation resource allocation on rural revitalization by constructing an econometric model. We found that the overall efficiency of the overall allocation of agricultural science and technology innovation resources in Anhui Province increased in the sample period, but the scale efficiency level was relatively low. The key path to improving the overall efficiency of allocation was to improve the scale efficiency level. The allocation of agricultural science and technology innovation resources in 16 cities and prefectures performed well in terms of pure technical efficiency, but there were significant differences in scale efficiency, which further affected the overall allocation efficiency of different regions. Among them, the allocation efficiencies of agricultural science and technology innovation resources in Hefei and Fuyang were at the leading level in Anhui Province. Similar to the overall situation of the province, the improvement path of areas with low comprehensive efficiency lay in the improvement of scale efficiency. In view of this, from the policy level, we need to optimize the relationship between the government and the market, speed up the construction of platforms and carriers, attach importance to the construction of the agricultural science and technology talent training system, and improve the open sharing mechanism.

## 1. Introduction

Green agriculture is an important cornerstone for realizing the overall health of people [1]. Agricultural science and technology innovation is related to the realization of green agriculture and is the key to the high-quality development of agriculture. The promotion of the green development of agriculture is a profound revolution in the concept of agricultural development. Practice has proven that in order to enhance the momentum of green agricultural development, we must take reform and innovation as the driving force and improve the system and mechanisms. The green development of agriculture requires the coordinated promotion of the agricultural production and operation mode, an agricultural spatial layout system, a green ecologically oriented policy support system, scientific and technological innovation, and a promotion system. Science and technology resources are strategic resources. The allocation of scientific and technological resources is a dynamic process of maximizing scientific and technological resources through adjustment and allocation [2]. In terms of overall progress, China’s research on the allocation of scientific and technological resources shows promising prospects for scale development, obvious structural diversity, room for quality improvement, and increasingly clear research trends. From the perspective of stage characteristics, they are distinct, and the differences are significant. The reform of the science and technology system has played a different role in the research of each stage [3].

The agricultural sector should increase investment in scientific and technological innovation for green agricultural development. Agricultural development should also improve the scientific and technological innovation system of green agricultural development, improve the diversified agricultural technology extension service system, and strengthen scientific and technological support for green agricultural development. In the new era, we will explore new mechanisms of scientific and technological innovation cooperation; increase investment in research and development of green agricultural production technologies such as the reduction and efficient use of agricultural inputs, agricultural water conservation, green pest prevention and control, and waste recycling; and accelerate the demonstration, promotion, and application of mature and applicable green technologies and green achievements. In terms of research on the allocation of scientific and technological innovation resources, foreign research on this aspect started early, with extensive and in-depth research [4,5,6,7,8]. A large amount of research has focused on a specific region in China, using different measurement tools to measure the allocation of scientific and technological innovation resources and analyze its influencing factors [9,10,11]. Another type research has focused on the comparative analysis between different regions. It has been found that there are significant regional differences in the allocation efficiency of scientific and technological innovation resources in China [12,13,14,15,16,17].

At present, China’s green agricultural development needs to make full use of and rely on green innovative technologies related to low cost, low energy consumption, low pollution, and low emissions in order to effectively promote green agricultural scientific and technological innovation and promote the transformation and upgrading of green agriculture. Green agricultural scientific and technological innovation is the specific performance of scientific and technological innovation in the specific field of agriculture. It is emphasized that, with the help of scientific and technological innovation, while improving the input–output efficiency, the intensity of carbon emissions and environmental pollution are effectively reduced. Focusing on green agricultural scientific and technological innovation, more research still centers itself on the measurement of green agricultural scientific and technological innovation resource allocation and the horizontal comparison of different regions. Some studies argue that the coordination level of China’s agricultural science and technology resource allocation ability subsystem and allocation efficiency subsystem is at the primary level and has not been effectively improved [18]. The gap in China’s agricultural science and technology resource allocation capacity is mainly due to regional differences [19]. Technical efficiency has become the main factor affecting the allocation efficiency of agricultural science and technology resources [20,21,22,23,24]. In addition, there are also sporadic research achievements in the analysis of the allocation mode of green agricultural science and technology innovation resources and the analysis and reference of the experience of foreign agricultural science and technology resource allocation, evaluation index design, and international comparison [25,26,27,28].

The previous research results provide ideas and methods for the development of this study. On the one hand, the mature use of various econometric models in calculating the allocation of agricultural science and technology innovation resources in the previous research results has provided an index for the selection of methods in this study; on the other hand, the research framework of the previous research results in vertical comparison of the same region and horizontal comparison of different regions, which was also adopted by this study. Moreover, the induction of the factors that affect the efficiency of agricultural science and technology innovation resource allocation provides inspiration for this study.

The key to China’s agricultural and rural modernization lies in the innovation of green science and technology. Green development is an important connotation of high-quality agricultural development. China is a large agricultural country. We should strengthen the promotion and application of modern agricultural science and technology; actively develop green agriculture, ecological agriculture, and efficient agriculture; and firmly put the Chinese people’s jobs in their own hands. High-quality economic growth is driven by the improvement of scientific and technological innovation [29]. Rural revitalization is the focus of China’s “three rural” work at this stage, and green agricultural science and technology innovation is the internal driving force to promote rural revitalization. Scientific and technological innovation is an important form of support for green agriculture, being at the core of improving agricultural competitiveness and an important way of promoting agricultural and rural modernization. Focusing on the allocation of green agricultural scientific and technological innovation resources and analyzing its allocation level will help in providing dynamic support for rural revitalization in China at the level of scientific and technological innovation, and thus, it has strong practical significance.

This study takes the Anhui Province of China as an example in order to accurately understand the allocation level of green agricultural science and technology innovation resources in this region by calculating the allocation efficiency, so as to provide a reference for its further optimization and promotion. The reason for choosing Anhui Province as the sample region lies in two aspects: First, we hoped to obtain relatively more targeted research findings from a small sample. China has a vast territory and significant differences between different regions. By narrowing the observation sample, we were able to effectively improve the pertinence of the research findings. Second, as a sample region, Anhui Province is very representative. On the one hand, Anhui Province is the birthplace of China’s rural reform and has been a major agricultural province for a long period of time. In 2020, its total output value of agriculture, forestry, animal husbandry, and fishery accounted for 4.12% of China’s total output value. On the other hand, Anhui Province is an integral province of the Yangtze River Delta, and also a core member of the Yangtze River Delta Science and Technology Innovation Community, which has the dual characteristics of agricultural and rural areas and scientific and technological innovation. Furthermore, the unique geographical characteristics of Anhui Province make it a research object. Anhui Province is divided into three regions by the Huaihe River and the Yangtze River, namely, the region to the north of the Huaihe River (Huaibei, Bozhou, Suzhou, Bengbu, Fuyang, Huainan), the region between the Yangtze River and the Huaihe River (Hefei, Chuzhou, Lu’an, Anqing), and the region to the south of the Yangtze River (Huangshan, Chizhou, Wuhu, Ma’anshan, Xuancheng, Tongling); the region to the north of the Huaihe River is a plain area, the area between the Yangtze River and the Huaihe River is a hilly area, and the area to the south of the Yangtze River is mostly a mountainous area. The dialects, customs, and economic development levels of the three regions are significantly different, forming three relatively independent human geographical units. This may lead to the unbalanced allocation of green agricultural scientific and technological innovation resources in different regions.

The shortcoming of this study is that it did not make a significant breakthrough in the use of research methods and the design of the overall analysis framework compared with existing research. The contribution of this study was to determine the allocation of agricultural science and technology innovation resources in Anhui Province, China, and to provide a reference for the region to improve the allocation efficiency in the future.

## 2. Materials and Methods

### 2.1. Model Construction

**Figure 1:**
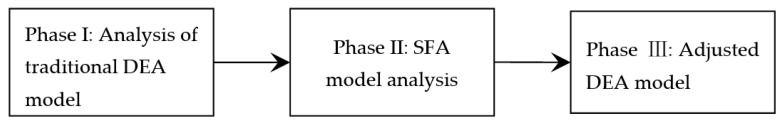
Flow chart of research methods.

This study used a three-stage DEA model to calculate the allocation of green agricultural science and technology innovation resources in Anhui Province (Figure 1).

#### 2.1.1. Phase I: Analysis of the Traditional DEA Model

In this paper, the BCC model (variable return to scale) of input orientation proposed by Banker et al. (1984) was selected to calculate the initial efficiency level of each DMU (decision-making unit) relative effective production frontier. This model can decompose the input–output efficiency (technical efficiency, TE) of each decision-making unit (DMU) into pure technical efficiency (PE) and scale efficiency (SE). The input–output efficiency (TE) refers to the ability of each DMU to achieve the maximum output under the given input conditions, the pure technical efficiency (PE) refers to the production efficiency of each DMU under the given management level and technical level, and the scale efficiency (SE) refers to the production efficiency of each DMU under the given production scale economy. The input–output efficiency is equal to the product of the pure technical efficiency and the scale efficiency (TE = PE × SE). The dual BCC model under input guidance can be expressed as:

(1) $$\mathrm{Min}\theta -\varepsilon ({\hat{e}}^{T}S^{-}+e^{T}S^{+})$$

(2) $$s.t.\left\{ \begin{array}{l} \sum_{i=1}^{n}X_{i}\lambda_{i}+S^{-}=\theta X_{0}\\ \sum_{i=1}^{n}Y_{i}\lambda_{i}-S^{+}=Y_{0}\\ \lambda_{i}\geq 0,S^{-}\geq \;0,S^{+}\geq \;0\\ i=1,2,3,\ldots ,n \end{array}\right.$$

where X is the input vector; Y is the output vector; S^−^ and S^+^ represent slack variables of input and output factors, representing input redundancy and output insufficiency, respectively; e*^T^* represents the unit row vector; and θ is the effective value of DMU.

#### 2.1.2. Phase II: SFA Model Analysis

In order to obtain the adjusted input variables by peeling off the impact of environmental factors and random disturbances on the allocation efficiency of green agricultural scientific and technological innovation resources, the SFA equation with the input relaxation value of the first stage as the dependent variable and the environmental variable as the explanatory variable was constructed in reference to the research of Fried et al. (2002) [30] as follows:

(3) $$\begin{array}{l} S_{nj}=f(Z_{j};\beta_{n})+\nu_{nj}+\mu_{nj}\\ i=1,2,\ldots ,I;n=1,2,\ldots ,N \end{array}$$

where S_nj_ represents the relaxation value of the nth input of the jth decision-making unit; Z_j_ represents the environment variable; β_n_ represents the estimation coefficient of the environment variable; ν_nj_ represents random interference, assuming that it follows a normal distribution $\nu_{\mathrm{nj}}{\sim N(0,\sigma }_{\nu }^{2})$, and represents the influence of random interference factors on the relaxation value; μ_nj_ indicates management inefficiency, assuming that it obeys the normal distribution truncated from zero point $\mu_{\mathrm{nj}}{\sim N}^{+}{(0,\sigma }_{\mu }^{2})$, indicating that management factors affect the relaxation value; and ν_nj_ and μ_nj_ are independent of each other, and are not related to Z_j_, ν_nj_ + μ_nj_, indicating a mixed error term.

Adjust the input variables of all DMUs according to the estimation results of the above formula:

(4) $$\begin{array}{l} X_{nj}^{A}=X_{nj}+\left[max(f(Z_{j};{\hat{\beta }}_{n}))-f(Z_{j};{\hat{\beta }}_{n})\right]+\left[max(\nu_{nj})-\nu_{nj}\right]\\ i=1,2,\ldots ,I;n=1,2,\ldots N \end{array}$$

where $X_{\mathrm{nj}}^{A}$ represents the adjusted input, and $x_{\mathrm{nj}}$ represents the investment before adjustment.

#### 2.1.3. Phase III: Adjusted DEA Model

The process of the third stage is similar to that of the first stage. The input variables adjusted in the second stage are used, and the BCC-DEA model of input orientation is used to recalculate the allocation efficiency of green agricultural scientific and technological innovation resources to obtain a new efficiency value of the decision-making unit. The efficiency value at this time is a relatively more true and accurate allocation efficiency value after excluding the impact of environmental factors and random errors.

### 2.2. Variable Selection

The input indicators used in this study included agricultural R&D personnel input and agricultural R&D funds input. The former is expressed by the full-time equivalent of agricultural R&D personnel, while the latter uses the internal expenditure of agricultural R&D funds. The output indicators used included patent output, paper output, and economic output. The patent output is expressed by the number of patent authorizations; the paper output is expressed by the number of scientific and technological papers published; and the economic output is expressed by the total output value of agriculture, forestry, animal husbandry, and fishery [31,32]. The specific types and meanings of variables are shown in Table 1.

In the second stage of SFA model calculation, on the basis of the consideration of representativeness, relevance, and availability, this study selected five variables as the external factors that affect the efficiency of green agricultural science and technology innovation resource allocation in various regions of Anhui Province, namely, trade openness, economic development level, higher education level, financial support, and Internet penetration, in order to eliminate the impact of environmental factors. The specific types and meanings of variables are shown in Table 2.

### 2.3. Data Source

Due to the limitation of the availability of relevant data, the sample period was set as 2010–2020. The sample areas used were 16 cities in Anhui Province. The data used in this research were from Anhui Statistical Yearbook and China Urban Statistical Yearbook. For the missing data in some years, the interpolation method was used to make up for them, and the relevant data were subject to price adjustment.

## 3. Results

### 3.1. Phase I: DEA Estimation Result Analysis

The first and third stages of DEA analysis were conducted using software Deap2.1. The first stage of DEA estimation preliminarily showed the efficiency of the allocation of green agricultural science and technology innovation resources in Anhui Province in the sample period. According to the estimated results, the overall performance of the allocation efficiency of agricultural science and technology innovation resources in Anhui Province was not outstanding, while the performance of 16 cities showed obvious heterogeneity.

From the perspective of the provincial average (Table 3), during 2010–2020, the allocation efficiency of green agricultural science and technology innovation resources in Anhui Province did not reach the pre-production line, and the overall efficiency showed a fluctuating trend, with the minimum value of 0.696 in 2016 and the maximum value of 0.785 in 2013. The values of pure technical efficiency and scale technical efficiency also showed the characteristics of fluctuation during the sample period, with the minimum values found in 2016 (0.789) and 2012 (0.793). On the whole, under the existing input conditions, there is still much room for growth and improvement in the efficiency of the allocation of green agricultural science and technology innovation resources in Anhui Province.

In terms of comprehensive efficiency by region (Table 4), in the sample period, only Suzhou City’s average comprehensive efficiency reached 1. The average comprehensive efficiencies of Bozhou, Fuyang, Huainan, Lu’an, and Chizhou were in the range of (0.9, 1); the average comprehensive efficiency of Hefei was in the range of (0.8, 0.9); the average comprehensive efficiencies of Bengbu and Anqing were in the range of (0.7, 0.8); the average comprehensive efficiencies of Huaibei, Chuzhou, Wuhu, Xuancheng, and Huangshan were in the range of (0.5, 0.7); and the average comprehensive efficiencies of Ma’anshan and Tongling were 0.489 and 0.325, respectively, which were in the last two places of all sample areas.

In terms of pure technical efficiency, the average pure technical efficiencies of Hefei, Suzhou, and Fuyang reached 1; the mean value of pure technical efficiencies of Bozhou, Huainan, Lu’an, Chizhou, and Huangshan were within the range of (0.9, 1); the mean values of pure technical efficiency of Huaibei and Bengbu were within the range of (0.8, 0.9); the average pure technical efficiencies of Chuzhou, Wuhu, Tongling, and Anqing were within the range of (0.7, 0.8); and the average pure technical efficiencies of Ma’anshan and Xuancheng were 0.535 and 0.653, respectively.

In terms of scale efficiency, only Suzhou’s average scale efficiency reaches 1. The average scale efficiencies of Bozhou, Fuyang, Huainan, Lu’an, Ma’anshan, Xuancheng, Chizhou, and Anqing were within the range of (0.9, 1); the average scale efficiencies of Hefei, Huaibei, Bengbu, Chuzhou, and Wuhu were within the range of (0.8, 0.9); and the average scale efficiencies of Huangshan and Tongling is 0.647 and 0.430, respectively, which were in the last two places of all sample areas.

### 3.2. Phase II: SFA Estimation Result Analysis

We used the software Frontier 4.1 to obtain the regression results of the second-stage SFA model (Table 5). According to the estimation results, the sigma squared, gamma, and LR unilateral tests of the two equations all passed the 1% significance test, indicating that the regression of SFA was effective, and the design of the two equations was reasonable and correct. It also indicates that the conclusions obtained from the DEA analysis using sample data without eliminating environmental effects were lack of authenticity and scientificity.

In the equation with the investment relaxation variable of agricultural R&D personnel as the dependent variable, the estimation coefficients of higher education level, financial support, and Internet penetration rate passed the significance test, showing that the above variables had a significant impact on the redundancy of the investment relaxation variable of agricultural R&D personnel. Among them, the increase in the number of full-time teachers in ordinary colleges and universities was conducive to improving the allocation efficiency of green agricultural science and technology innovation resources in Anhui Province, while the increase in financial support and Internet penetration will increase the redundant variable of agricultural R&D personnel investment.

In the equation with the relaxation variable of internal expenditure of R&D funds as the dependent variable, the estimated coefficients of environmental variables passed the 1% significance test, having a significant impact on the redundant variables of agricultural R&D funds. The estimated coefficients of the import and export ratio, the economic development level, and the higher education level were negative. The increase in the above three variables will help reduce the redundant variables of agricultural R&D investment, thus improving the efficiency of green agricultural scientific and technological innovation resource allocation in Anhui Province. The estimated coefficient of financial support and Internet penetration rate was positive, showing a significant “crowding out effect”. The increase will lead to a waste of agricultural R&D funds, which is unfavorable for the improvement of resource allocation efficiency of green agricultural scientific and technological innovation.

### 3.3. Phase III: DEA Estimation Result Analysis

The second stage SFA regression results were used to adjust the original input variables. After eliminating the influence of interference factors, the input indicators and the original output indicators were analyzed by the BCC-DEA model again to calculate the real efficiency value of green agricultural scientific and technological innovation resource allocation. From the results, the estimated value in the third stage was adjusted in comparison with that in the first stage, but the overall performance of the allocation efficiency of agricultural science and technology innovation resources in Anhui Province still showed a large improvement and space for further improvement, whereas the performance of the 16 cities was uneven.

From the perspective of the provincial average value (Table 6), the adjusted comprehensive efficiency of green agricultural science and technology innovation resource allocation in Anhui Province showed an overall increasing trend in the sample period, growing from 0.623 in 2010 to 0.707 in 2020. Its growth was mainly driven by the improvement of scale efficiency. Although the pure technical efficiency fluctuated slightly in the sample period, the overall situation remained relatively stable, with small changes around 0.995. In the sample period, the scale efficiency showed an upward trend in fluctuations, rising from the minimum value of 0.623 in 2010 to the maximum value of 0.718 in 2020.

Although the third stage DEA estimation results were more consistent with the actual development of Anhui Province in recent years, the maximum values of comprehensive efficiency and scale efficiency did not exceed 0.800, which means that improving the scale efficiency level was the key path to improving the allocation of green agricultural science and technology innovation resources in Anhui Province at this stage.

By region (Table 7), in terms of comprehensive efficiency, the average comprehensive efficiencies of Hefei and Fuyang reached 1, which was along the line before production and ranked the top among all sample regions; the second was Suzhou and Lu’an, with the average values of comprehensive efficiency within the range of (0.9, 1); the third was Bozhou, Bengbu, Chuzhou, Wuhu, and Anqing, with the average values of comprehensive efficiency within the range of (0.8, 0.9); the average comprehensive efficiencies of Huainan and Xuancheng were within the range of (0.5, 0.7); the average comprehensive efficiencies of Huaibei, Maanshan, Tongling, Chizhou, and Huangshan were within the range of (0.1, 0.5), among which the average comprehensive efficiencies of Huangshan and Tongling were only 0.250 and 0.152, respectively, ranking as the last two in all sample areas.

In terms of pure technical efficiency, each sample area showed a good trend, with an average value of 1 in Hefei, Bozhou, Suzhou, Fuyang, Lu’an, Tongling, Chizhou, and Huangshan; the average pure technical efficiencies of Huaibei, Bengbu, Huainan, Chuzhou, Maanshan, Wuhu, Xuancheng, and Anqing were within the range of (0.9, 1).

In terms of scale efficiency, the sample regions showed obvious differences, and the average scale efficiencies of Hefei and Fuyang were both 1; the second place was Suzhou, Chuzhou, Lu’an, and Wuhu, with average scale efficiencies within the range of (0.9, 1); thirdly was Bozhou, Bengbu, and Anqing, with average scale efficiencies within the range of (0.8, 0.9); the average scale efficiencies of Huainan and Xuancheng were within the range of (0.5, 0.7); the average scale efficiencies of Huaibei, Maanshan, Tongling, Chizhou, and Huangshan were within the range of (0.1, 0.5), among which the average scale efficiencies of Huangshan and Tongling were 0.250 and 0.152, respectively, at the bottom of all sample areas.

We further classified the sample regions according to the third-stage DEA estimation results (Table 8). On the whole, the allocation level of green agricultural science and technology innovation resources in Anhui Province showed obvious differences among the 16 cities, among which the allocation level in the north of the Huaihe River and between the Yangtze River and the Huaihe River was relatively good, while the allocation level in the south of the Yangtze River was relatively low.

Specifically, Hefei and Fuyang belong to the regions with excellent configuration, while Bozhou, Suzhou, Bengbu, Chuzhou, Lu’an, Wuhu, and Anqing belong to the regions with good configuration; Huaibei, Huainan, Ma’anshan, Xuancheng, Tongling, Chizhou, and Huangshan belong to the group that requires improvement in scale efficiency. Due to the low scale efficiency, these regions have low efficiency in allocating green agricultural scientific and technological innovation resources. Therefore, the focus of future optimization is to improve scale efficiency.

## 4. Discussion and Conclusions

### 4.1. Conclusions

This research focused on the analysis of resource allocation efficiency of green agricultural science and technology innovation. Taking Anhui Province, the birthplace of China’s rural reform, as the object of focus, by building a three-stage DEA model, we calculated the resource allocation efficiency of green agricultural science and technology innovation in this region from 2010 to 2020. The research made the following findings:

From the perspective of the whole province, the overall efficiency of the allocation of green agricultural science and technology innovation resources in Anhui Province increased in the sample period, but the level of scale efficiency was relatively low. The key way to improve the overall efficiency of allocation is to improve the level of scale efficiency.

From the perspective of each region, the allocation of green agricultural science and technology innovation resources in 16 cities in Anhui Province performed well in terms of pure technical efficiency, but there were significant differences in scale efficiency, which further affected the overall allocation efficiency of each region. Among them, the allocation efficiency of green agricultural science and technology innovation resources in Hefei City and Fuyang City was at the leading level in Anhui Province. Similar to the overall situation of the province, the improvement path of areas with low comprehensive efficiency lay within the improvement of scale efficiency.

The findings of this study not only clarify the overall level of the allocation of agricultural science and technology innovation resources in Anhui Province and the specific conditions of 16 regions but also provide a reference for later research. On the basis of the findings of this study, further research on the influencing factors of allocation, resource allocation, and rural revitalization can be extended. Although the analysis of this study was carried out within a mature research framework, due to the lack of accurate statistical data on agricultural scientific and technological innovation, the weight index was used for corresponding processing. Although this method is feasible, it is undeniable that it will have a certain impact on the accuracy of the calculation results, which needs to be improved and perfected in subsequent studies.

### 4.2. Policy Implications

#### 4.2.1. Optimize the Relationship between the Government and the Market

We should focus on accelerating the market construction of green agricultural scientific and technological innovation factors from the aspects of factor flow barriers, factor price rigidity, and factor price differentiation, so that the market can play a leading role in the allocation of scientific and technological resources while giving full play to the role of the government in the overall planning, coordination, supervision, and regulation of scientific and technological resource allocation, as well as further improving and balancing the relationship between the government and the market in resource allocation.

#### 4.2.2. Accelerate the Construction of Platform and Carrier

First, we should increase the application and construction of national agricultural high-tech industry demonstration zones, national agricultural science and technology parks, and provincial agricultural science and technology parks. Second, we should speed up the construction process of innovation carriers such as agricultural and forestry comprehensive stations and agricultural technology extension demonstration bases. Third, we should innovate the service mode of agricultural science and technology extension and accelerate the transformation of agricultural scientific and technological achievements.

#### 4.2.3. Attach Importance to the Construction of an Agricultural Science and Technology Personnel Training System

Agricultural science and technology talents are the soul of green agricultural science and technology innovation resource allocation. On the one hand, we should focus on the cultivation of individual agricultural science and technology talents to improve the individual scientific research level; on the other hand, we should pay more attention to the construction of the talent system, build a reasonable talent training system, and provide intellectual resources for the allocation of green agricultural scientific and technological innovation resources.

#### 4.2.4. Improve the Open Sharing Mechanism

Under the guidance of informatization, standardization, and networking, we must further improve the open mechanism of green agricultural scientific and technological innovation resources, effectively realize multi-dimensional sharing of agricultural scientific and technological resources, improve the management mechanism and relevant laws and regulations of scientific and technological resource sharing, change the problems of duplication and fragmentation of green agricultural scientific and technological innovation resources at this stage, and improve the use efficiency of scientific and technological resources.

## Figures and Tables

**Table 1:** Input–output indicators of the DEA model.

Type	Variable	Variable Definition
Input index	Agricultural R&D personnel input	Full-time equivalent of agricultural R&D personnel
	Agricultural R&D funds input	Internal expenditure of agricultural R&D funds
Output index	Patent output	Number of patents granted
	Paper output	Number of published scientific papers
	Economic output	Gross output value of agriculture, forestry, animal husbandry and fishery

**Table 2:** Environmental variables of the SFA model in the second stage.

Type	Variable	Variable Definition
Dependent variable	Input relaxation variable	Relaxation value of input variables of each decision unit
Independent variable	Trade openness	Total import and export/regional GDP
	Level of economic development	Per capita GDP
	Higher education level	Number of full-time teachers in colleges and universities
	Financial support	Financial expenditure on science and technology/financial expenditure
	Internet penetration	Broadband access number

**Table 3:** DEA estimation results of the first stage: average of the whole province.

Time	Comprehensive Efficiency	Pure Technical Efficiency	Scale Efficiency
2010	0.766	0.853	0.899
2011	0.770	0.906	0.856
2012	0.704	0.899	0.793
2013	0.785	0.907	0.840
2014	0.743	0.920	0.813
2015	0.746	0.840	0.894
2016	0.696	0.789	0.871
2017	0.759	0.819	0.922
2018	0.764	0.830	0.912
2019	0.784	0.821	0.954
2020	0.698	0.818	0.854

**Table 4:** DEA estimation results of the first stage: regional average.

Serial Number	Region	Comprehensive Efficiency	Pure Technical Efficiency	Scale Efficiency
1	Hefei	0.837	1.000	0.837
2	Huaibei	0.666	0.821	0.809
3	Bozhou	0.995	0.998	0.996
4	Suzhou	1.000	1.000	1.000
5	Bengbu	0.755	0.847	0.894
6	Fuyang	0.958	1.000	0.958
7	Huainan	0.903	0.922	0.980
8	Chuzhou	0.567	0.731	0.823
9	Lu’an	0.978	0.988	0.991
10	Maanshan	0.489	0.535	0.914
11	Wuhu	0.568	0.722	0.834
12	Xuancheng	0.636	0.653	0.966
13	Tongling	0.325	0.793	0.430
14	Chizhou	0.919	0.999	0.921
15	Anqing	0.703	0.720	0.975
16	Huangshan	0.611	0.961	0.647

**Table 5:** Regression results of SFA in the second stage.

Variable	Relaxation Variables of Agricultural R&D Personnel Input		The Slack Variable of Agricultural R&D Investment	
	Coefficient of Regression	Standard Error	Coefficient of Regression	Standard Error
constant term	424.120 ***	118.599	8023.316 ***	1.480
Import and export degree	127.995	432.481	−4526.784 ***	1.113
Level of economic development	−2.643	49.705	−157.773 ***	1.823
Higher education level	−489.459 ***	94.158	−14,266.387 ***	1.207
Financial support	8851.221 ***	17.652	30.830 × 10^4^ ***	1.000
Internet penetration	1.5609 *	0.801	85.598 ***	1.553
sigma-squared	224,451.3 ***	1.683	212.656 × 10^6^ ***	1.000
gamma	0.458 ***	0.061	0.482 ***	0.059
Logarithmic likelihood value	−1294.028		−1892.063	
Unilateral test value	18.995 ***		21.082 ***	

Note: The upper corner marks * and *** indicate *p* < 0.1 and *p* < 0.01.

**Table 6:** DEA estimation results of the third stage: average of the whole province.

Time	Comprehensive Efficiency	Pure Technical Efficiency	Scale Efficiency
2010	0.623	0.998	0.623
2011	0.676	0.998	0.677
2012	0.697	0.998	0.698
2013	0.692	0.998	0.693
2014	0.699	0.997	0.700
2015	0.678	0.995	0.682
2016	0.679	0.988	0.688
2017	0.691	0.992	0.697
2018	0.662	0.997	0.664
2019	0.663	0.991	0.670
2020	0.707	0.987	0.718

**Table 7:** DEA estimation results in the third stage: regional average.

Serial Number	Region	Comprehensive Efficiency	Pure Technical Efficiency	Scale Efficiency
1	Hefei	1.000	1.000	1.000
2	Huaibei	0.308	0.990	0.311
3	Bozhou	0.802	1.000	0.802
4	Suzhou	0.988	1.000	0.988
5	Bengbu	0.881	0.991	0.888
6	Fuyang	1.000	1.000	1.000
7	Huainan	0.505	0.999	0.505
8	Chuzhou	0.889	0.973	0.914
9	Lu’an	0.911	1.000	0.911
10	Maanshan	0.445	0.990	0.449
11	Wuhu	0.891	0.988	0.901
12	Xuancheng	0.671	0.990	0.677
13	Tongling	0.152	1.000	0.152
14	Chizhou	0.360	1.000	0.360
15	Anqing	0.807	0.992	0.813
16	Huangshan	0.250	1.000	0.250

**Table 8:** Regional classification according to allocation level.

Type	Classification Standard	Region
Excellent configuration type	Pure technical efficiency = 1 Scale efficiency = 1	Hefei, Fuyang
Good configuration type	0.9 ≤ pure technical efficiency < 1 0.75 ≤ Scale efficiency < 1	Bozhou, Suzhou, Bengbu, Chuzhou, Lu’an, Wuhu and Anqing
Scale efficiency improvement	0.9 ≤ pure technical efficiency < 1 0 ≤ Scale efficiency < 0.75 0 ≤ Scale efficiency < 0.75	Huaibei, Huainan, Maanshan, Xuancheng, Tongling, Chizhou and Huangshan

## Data Availability

The data presented in this study are available on request from the corresponding author. The data are not publicly available due to personal privacy and non-open access to the research program.

## References

[1] Xu X., Wang Q., Li C. (2022). The Impact of Dependency Burden on Urban Household Health Expenditure and Its Regional Heterogeneity in China: Based on Quantile Regression Method. Front Public Health.

[2] Bronzini R., Piselli P. (2016). The Impact of R&D Subsidies on Firm Innovation. Res. Policy.

[3] Gao J., Yue W., Suo W. (2018). S&T Resource Allocation Research Progress and Phased Policy Influence. Manag. Rev..

[4] Wakelin K. (2001). Productivity Growth and R&D Expenditure in UK Manufacturing Firms. Res. Policy.

[5] Rafferty M., Funk M. (2004). Demand Shocks and Firm-financed R&D Expenditures. Appl. Econ..

[6] Bellais R. (2004). Post Keynesian Theory, Technology Policy, and Long-term Growth. J. Post Keynes. Econ..

[7] Falk M. (2012). Quantile Estimates of the Impact of R&D Intensity on Firm Performance. Small Bus. Econ..

[8] Ugur M., Trushin E., Solomon E., Guidi F. (2016). R&D and Productivity in OCED Firms and Industries: A Hierachical Meta-Regression Analysis. Res. Policy.

[9] Li Y., Bai L. (2019). Study on Resource Allocation Efficiency and Influencing Factors of Agricultural Science and Technological Innovation in Yunnan Province. Chin. J. Agric. Resour. Reg. Plan..

[10] Zhao L. (2018). Study on Allocation Efficiency and Influencing Factors of Agricultural Science and Technology Innovation Resources in Chongqing. Chin. J. Agric. Resour. Reg. Plan..

[11] Yue H., Sun C. (2019). Analysis of Agricultural Technology Innovation Efficiency and Influencing Factors in Shandong Province Based on DEA-Tobit Model. IOP Conf. Ser. Mater. Sci. Eng..

[12] Ma Y., Ma Y. (2021). Study on Regional Differences and Convergence of the Allocation Efficiency of Scientific and Technological Resources in China. J. Quant. Tech. Econ..

[13] Duan Z., Wu P. (2021). Research on the Configuration and Path of the Influencing Factors of the Allocation Efficiency of Science and Technology Resources: QCA Analysis Based on 30 Provinces in China. Sci. Tchnol. Prog. Policy.

[14] Liu Z., Chen J. (2019). Evaluation of the Allocation Level of Science and Technology Resources and Regional Differences. Sci. Technol. Manag. Res..

[15] Liang L., Li Q., Liu B. (2019). The Efficiency of Scientific and Technological Resources Allocation in China’s Provinces under Environmental Constraint: Spatiotemporal Pattern, Eovlution Mechanism and Influencing Factors. Forum Sci. Technol. China.

[16] Fan J., Zhao Y., Wu M. (2017). Chinese Science and Technology Innovation Resources Allocation Based on the Improved Cross-Efficiecy Method. Forum Sci. Technol. China.

[17] Liang X., Wang X., Gong Q., Li S. Research on the efficiency of chinese agricultural science and technology innovation based on the lagging dea model. Proceedings of the 4th International Conference on Computers in Management and Business.

[18] Ding L., Li H., Yang C. (2021). The Spatial-temporal Differentiation and Driving Force of the Coupling Coordination of Agricultural S & T Resource Allocation System in China. Sci. Manag. Res..

[19] Chen Q., Zhang J., Cheng L., Li Z. (2016). Regional Difference Analysis and Its Driving Factors Decomposition on the Allocation Ability of Agricultural Science and Technology. Sci. Res. Manag..

[20] Deng M., Yang C. (2017). Analysisi on the Dynamic Evolution of Agricultural Science and Technology Resources Allocation Efficiency Based on Super Efficiency DEA Model. Chin. J. Agric. Resour. Reg. Plan..

[21] Zhang Y., Wang M., Wang X. (2018). Evaluation of Agricultural Scientific and Technological Resources Allocation Efficiency and Its Promotion Countermeasures: A Case Study of Hubei Province. J. Shangdong Univ. Financ. Econ..

[22] Yang C., Wang Y., Xu W., Zhang J. (2016). Study on Structural Effect of Agriculture S & T Resources Allocation Based on Computational Experiment. Sci. Technol. Prog. Policy.

[23] Ji H., Wei J. (2020). Research on the Efficiency of Agricultural S & T Innovation Resource Allocation for Rural Revitalization: Case Study of Zhejiang Province. Sci. Technol. Dev..

[24] Wang Y., Yang C., Tao Q. (2018). Research on Evaluation and Promotion of Agricultural Scientific and Technical Resource Allocation Ability in Ethnic Minority Areas. China Sci. Technol. Resour. Rev..

[25] Yang C., Zhang J. (2012). Expericence Analyzing of Agricultural Science and Technology Resources Allocation About American and Japanese. Agric. Econ. Manag..

[26] Jiang M., Yu M., Li H. Construction and application of evaluation index system of agricultural scientific and technological progress under the background of modern agriculture—An empirical research based on statistical data of Ningbo City. Proceedings of the 2014 International Conference on Management Science & Engineering 21th Annual Conference Proceedings.

[27] Guo X., Deng C., Wang D., Du X., Li J., Wan B. (2021). International comparison of the efficiency of agricultural science, technology, and innovation: A case study of G20 countries. Sustainability.

[28] Xiao W. (2020). Technological Progress and the Transformation of China’s Economic Development Mode.

[29] Romer P.M. (1990). Endogenous Technological Change. J. Political Econ..

[30] Fried H., Lovell C., Schmidt S. (2002). Accounting for Enovironmental Effects and Statistical Noise in Data Envelopment Analysis. J. Product. Anal..

[31] Wang F., Liu Y. (2022). Agricultural Science and Technology Innovation Efficiency and Regional Difference Comparison in China: Based on the Perspective of Grain Functional Areas. Sci. Technol. Ind..

[32] Chen Z., Zheng R., Li P., Huang S. (2018). Evaluation and Analysis of Agricultural Science and Technology Innovation Efficiency in Henan Province. J. Henan Agric. Univ..

